# The state and consideration for skin test of β-lactam antibiotics in pediatrics

**DOI:** 10.3389/fcimb.2023.1147976

**Published:** 2023-06-15

**Authors:** Chunhui Gao, Bowen Ma, Wei Liu, Liqin Zhu

**Affiliations:** ^1^ Department of Pharmacy, Tianjin Children's Hospital (Children's Hospital of Tianjin University), Tianjin, China; ^2^ Department of Pharmacy, Cangzhou People's Hospital, Cangzhou, Hebei, China; ^3^ Tianjin Children's Hospital (Children's Hospital of Tianjin University), Tianjin, China; ^4^ Department of Pharmacy, Tianjin First Central Hospital, Tianjin, China

**Keywords:** β-lactam antibiotics, anaphylaxis, skin test, drug provocation test, pediatrics

## Abstract

β-lactam antibiotics are the most frequently used drugs and the most common drugs that cause allergic reactions in pediatrics. The occurrence of some allergic reactions can be predicted by skin testing, especially severe adverse reactions such as anaphylactic shock. Thus, penicillin and cephalosporin skin tests are widely used to predict allergic reactions before medication in pediatrics. However, false-positive results from skin tests were more often encountered in pediatrics than in adults. In fact, many children labeled as allergic to β-lactam are not allergic to the antibiotic, leading to the use of alternative antibiotics, which are less effective and more toxic, and the increase of antibiotic resistance. There has been controversy over whether β-lactam antibiotics should be tested for skin allergies before application in children. Based on the great controversy in the implementation of β-lactam antibiotic skin tests, especially the controversial cephalosporin skin tests in pediatrics, the mechanism and reasons of anaphylaxis to β-lactam antibiotics, the significance of β-lactam antibiotic skin tests, the current state of β-lactam antibiotic skin tests at home and abroad, and the problems of domestic and international skin tests were analyzed to determine a unified standard of β-lactam antibiotic skin tests in pediatrics to prevent and decrease adverse drug reactions, avoid waste of drugs, and a large amount of manpower and material resource consumption.

## 1 Introduction

β-lactam antibiotics are the most frequently used drugs and the most common drugs that cause allergic reactions in pediatrics; death could occur in severe cases due to anaphylactic shock. Penicillins and cephalosporins are the two main and most used β-lactam antibiotics, especially in children (Mori et al., 2019). A β-lactam antibiotic skin test is widely used to predict anaphylactic reactions before medication in pediatrics (Azevedo et al., 2019). However, most patients with suspected hypersensitivity reactions to β-lactam antibiotics could tolerate antibiotics. Positive results of skin tests were more often encountered in pediatrics than in adults (Graham et al., 2018). There has been controversy over whether β-lactam antibiotics should be tested for skin allergy before medication in children and a lack of unified standards and guidelines in the clinical operation of β-lactam antibiotic skin tests.

The primary aim of this review was to determine whether β-lactam antibiotics should be tested for skin allergy before application in children by analyzing the mechanism and reasons for anaphylaxis to β-lactam antibiotics, the significance of β-lactam antibiotic skin tests, the current state of β-lactam antibiotic skin tests at home and abroad, and the problems of domestic and international skin tests.

## 2 The mechanism of β-lactam antibiotics allergic reactions

### 2.1 The mechanism of drug hypersensitivity reactions

Drug hypersensitivity reactions (DHRs) are mediated by the immune system after exposure to drugs. Based on immunologic mechanisms, the Gell and Coombs classification divides them into four categories. Type I (immediate hypersensitivity) is mediated by IgE specific for allergens and occurs usually within a few minutes to an hour after administration; typical clinical manifestations include urticaria, angioneurotic edema, bronchospasm, and anaphylactic shock. Type II is characterized by antigen–antibody interactions, of which the vasculitides are classic examples. Type III is mediated by immune complexes, whose typical clinical manifestations include serum disease and drug-associated vasculitis. The clinical manifestations of Type IV hypersensitivity reactions, mediated by T cells, include eosinophilia, systemic symptom syndrome, Stevens–Johnson syndrome, and so on (Rajan, 2003; Dispenza, 2019; Wilkerson, 2022).

β-lactam antibiotic reactions are defined as immediate reactions (IR) or non-immediate reactions (NIR) based on the time interval from the last dose to the onset of symptoms (Romano et al., 2011; Demoly et al., 2014). IR occurs within 1 h after the last dose administration. The clinical manifestations of IR include urticaria and severe anaphylaxis (Demoly et al., 2014; Romano et al., 2020). NIR occurs more than one hour after the last dose administration and up to several hours or days (Romano et al., 2011). The clinical manifestations of NIR include urticaria, angioedema, and maculopapular exanthema.

### 2.2 The mechanism of penicillin allergy

The chemical structure of penicillin contains a β-lactam ring, a tetrahydrothiazole ring, and an R side chain. *In vivo*, the products of penicillin metabolism bind to self-proteins, resulting in allergic reactions (Lteif and Eiland, 2019). The reactive products of penicillin metabolism, also termed antigenic determinants, are classified into major and minor determinants. Benzyl penicilloyl (95%) is considered the major determinant, and other products (5%) include penicilloate, penicillanyl, and penicillenate, and others are considered minor determinants (Chang et al., 2012). It is the basic principle of skin testing and avoiding administration if a severe anaphylactic IgE reaction is observed.

The major determinant (benzylpenicilloyl polylysine, PPL) is recommended as the ideal skin test reagent. The most significant determinants include benzylpenicillin (penicillin G), benzylpenicilloate, and benzylpenilloate, as well as ampicillin or amoxicillin (Joint Task Force on Practice et al., 2010; Iammatteo et al., 2021), but there are no standardized reagents that contain all major and minor penicillin determinants commercially (Solensky et al., 2019).

### 2.3 The mechanism of cephalosporins allergy

The chemical structure of cephalosporins contains β-lactam ring, a six-membered dihydrothiazine ring, and R1 and R2 side chains, which differ from penicillins in the six-membered dihydrothiazine ring and R2 side chain. During the degradation of cephalosporins, the β-lactam ring, dihydrothiazine ring, and R2 side chain were disrupted, while the R1 side chain may remain undamaged. Unlike penicillins, for which the antigenic determinants are definite, the antigenic determinants of cephalosporins have not been clear and definite. In addition, cephalosporins’ efficiency in forming hapten protein conjugates is inefficient compared to penicillin. Some evidence supports the idea that the degradation of the β-lactam ring destroys the R2 side chain, resulting in unstable conjugates and deficiently identified determinants. The remaining β-lactam moiety and R1 side chain, which can link to host proteins covalently, are central to immune and allergic reactions (Khan et al., 2019).

### 2.4 Cross-reactivity in β-lactam allergy

The structure of all β-lactam antibiotics includes β-lactam ring, and the structure of penicillin has a thiazolidine ring. Different side chains distinguish different penicillins. Unlike the thiazolidine ring of penicillins, cephalosporins have a dihydrothiazine ring and R1 and R2 side chains, which distinguish different cephalosporins (Romano et al., 2018). During the drug metabolism of cephalosporins, the R1 side chain may remain intact, which can induce cross-reactivity with penicillins. Some evidence supports the idea that cross-reactivity between penicillins and cephalosporins primarily depends on whether their R1 side chain have a similar structure rather than the similarity of the β-lactam ring (Pichichero and Zagursky, 2014).

A meta-analysis of studies (Pichichero and Casey, 2007) indicated that first-generation cephalosporins increased anaphylactic reactions significantly, while there was no increase with second- and third-generation cephalosporins. According to a review of the cross-reactivity of β-lactam antibiotics with anaphylactic reactions (Zagursky and Pichichero, 2018). The prevalence of cross-reactivity between penicillins and cephalosporins was rare, and the occurrence of cross-reactivity was due to the similar structure of the R1 side chain. Patients with anaphylactic reactions to penicillins could be treated by administration of cefuroxime and ceftriaxone, whose side chains differ from those of penicillins (Romano et al., 2018). In addition, prospective studies demonstrated that cross-reactivity of penicillins and cephalosporins with monobactams and carbapenems was scarce (Gaeta et al., 2015; Mirakian et al., 2015; Romano et al., 2016; Zagursky and Pichichero, 2018), except for ceftazidime, which had the same R1 side chain as aztreonam (Frumin and Gallagher, 2009).

## 3 The significance of skin test

### 3.1 Penicillin skin test

Approximately 5% of children report a history of penicillin allergy. However, only a minority of these children were allergic. Due to the fact that penicillin allergy history had a poor prediction of reactivity, skin testing was key to identifying whether patients could be treated with penicillin safely (Picard et al., 2014). The penicillin skin test was the fastest, most sensitive, and most economical method to predict penicillin type I allergic reactions in children (Kulhas Celik et al., 2020). The standard penicillin skin test has a negative predictive value of 97%–99%, and reagents include major determinants, minor determinants, positive controls, and negative controls. Due to the lack of availability of major and minor determinant test reagents, the penicillin skin test is usually performed with diluted penicillin G (Geng et al., 2017).

### 3.2 Cephalosporin skin test

Unlike penicillins, where the antigenic determinants are stable and definite (Ariza et al., 2015; Khan et al., 2019), anaphylaxis to cephalosporins may occur due to unique antigenic determinants of cephalosporins or antigenic determinants that are shared with other β-lactam antibiotics infrequently, particularly penicillins. Given this reason, parent drugs are recommended as skin test reagents in addition to the classic benzylpenicillin reagents and semisynthetic penicillins (Romano et al., 2021). Although the cephalosporin skin test was less valuable than the penicillin skin test and had not been well validated, it had a good negative predictive value with different R1 side chains of cephalosporins. The ideal concentration for the cephalosporin skin test reagent has not been apparent strictly, and the association of the negative predictive value of the skin test with immediate hypersensitivity is uncertain (Khan et al., 2019). There were few research data available on the predictive values of skin tests for cephalosporins (Hershkovich et al., 2009).

## 4 The state of skin test in pediatrics

The routine skin test was not required before using β-lactam antibiotics in European and American countries; it was only carried out in China. In China, routine skin tests for cephalosporins had been canceled, but penicillin skin tests were still carried out at present for both adults and children. If penicillin was stopped for more than 72 h, the skin test should be repeated (Joerg et al., 2021; Jiang et al., 2023). In European and American countries, penicillin skin tests were only performed on patients with a history of allergies who needed penicillin (Forrest et al., 2001; Mirakian et al., 2015).

Since few studies have been performed on children, skin testing in the pediatric population has not been standardized. The guidelines, which can diagnose drug allergies in adults, were generally applied to pediatrics (Ibáñez et al., 2018). When the results of the skin test are positive, the patients are hypersensitive to the tested drug, and the administration is suspended (Kulhas Celik et al., 2020). In the past several years, the accuracy of skin tests has been questioned and discussed in some studies (Caubet et al., 2011; Ibáñez et al., 2018; Sousa-Pinto et al., 2021), and these studies highlighted that the diagnostic value of skin tests was not optimal in children. There are many diagnostic shortages in skin tests in children, such as low sensitivity and positive predictive value (PPV), especially for mild skin reactions (Arıkoğlu et al., 2022). A study indicated that skin tests could be false positives in 80% of cases, leading to the unnecessary avoidance of drugs (Ibáñez et al., 2018). A study indicated that higher concentrations of reagent, large injection volumes, and hidden additives or irritant effects could lead to false-positive results (Anterasian and Geng, 2018). In addition, due to the personal characteristics of the pediatricians, discomfort often occurred during the process of skin testing, which led to the expansion of the redness area. Skin tests in pediatrics, similar to adult studies, show a high negative predictive value (NPV), but a positive result might prevent the use of drugs because some studies confirmed a higher rate of false positives (Macy and Ngor, 2013; Vyles et al., 2017b; Solensky et al., 2019). The positive result of a skin test was still used to diagnose anaphylaxis in clinical practice, despite some reports of a low PPV of skin tests in children (Caubet et al., 2011; Ibáñez et al., 2018; Plager et al., 2021). In addition, low-efficiency, resource-intensive, and painful methods may limit the use of skin tests in children (Arıkoğlu et al., 2022), as a study indicated that the prescription costs were much higher in patients with labeled penicillin allergies (Norton et al., 2018).

Although the negative predictive value (NPV) of skin tests is high in both children and adults, some patients can experience an anaphylactic reaction after a negative result (Kulhas Celik et al., 2020). Two studies (Ibáñez et al., 2018; Labrosse et al., 2020) investigated the mild immediate and nonimmediate reactions to amoxicillin in children. There was a significant false-negative rate with the standard penicillin skin test in children. In infants and young children, skin reactivity is poor, and false-negative results may occur. In addition, some drugs can suppress anaphylactic reactions, leading to false negative results. We need to make sure of our medication history before a skin test.

Relatively few studies have evaluated the sensitivity and specificity of cephalosporin skin tests in patients with allergic reactions to cephalosporins. The prediction value of the cephalosporin skin test before administration in anaphylaxis is not supported by sufficient evidence-based medical evidence (Romano et al., 2010). Although conventional skin testing before administration of cephalosporins is not recommended, skin testing should be done in the following cases: Patients with a specific history of type I (immediate) allergy reactions to penicillin or cephalosporin, if it is necessary to use cephalosporins clinically for the patients, after obtaining the informed consent of the patient, should choose a cephalosporin with a side chain different from that of the allergy drug and the skin test results have certain reference values. Skin testing should be done when it is required in drug instructions (Kelkar and Li, 2001; Guéant et al., 2006; Brockow et al., 2013).

Skin testing is a painful method and difficult to interpret for children, especially infants. A false-positive result may increase the number of children suspected of having allergies to limit the use of antibiotics. The accuracy of skin tests in the allergic evaluation of suspected β-lactam allergic reactions has been highly debated recently (Moral and Caubet, 2017). In patients with suspected β-lactam antibiotic allergy reactions, non-β-lactam drugs, or desensitization are commonly used when alternative medicine is unavailable. Unfortunately, drug-resistant, resource-wasting, less effective, and more adverse reactions may occur when using alternative medicine or broad-spectrum antimicrobial agents, so all patients suspected of β-lactam allergy should be evaluated carefully.

## 5 More accurate allergy tests at present

### 5.1 Drug provocation test

Drug provocation test (DPT) is the method of administering a drug under controlled conditions to confirm whether there is an allergic reaction to the drug and whether the patient can tolerate the drug or not. The current data emphasize the accuracy of direct DPT in children with NIR and even potentially with IR, which is considered low risk. In some studies, only 3.4%–14% of children with a history of mild NIR had positive DPT and mild reactions. It is increasingly reported that direct DPT in children with a history of mild IR to β-lactam may be safe (Arıkoğlu et al., 2022). Accurate diagnosis of β-lactam anaphylactic reactions in children is often based on DPT (Garvey and Savic, 2019). In the last few years, direct DPT procedures without prior skin testing have gained acceptance as a safe and accurate strategy for patients (Caubet et al., 2011; Mill et al., 2016; Moral and Caubet, 2017; Macy and Vyles, 2018). According to the international consensus guidelines, skin testing is recommended as a first-line test for immediate reactions to drug allergies. If the result of the skin test is negative, DPT, as the current gold standard for diagnosis, is performed to confirm or exclude the presence of an allergy to the drug (Mirakian et al., 2015; Gomes et al., 2016; Romano et al., 2020), although no standardized protocols exist so far (Iammatteo et al., 2021).

Multiple studies supported the use of direct DPT without prior skin testing for pediatric and adult populations who were historically labeled with anaphylaxis to β-lactam antibiotics (Arıkoğlu et al., 2022). Serious adverse events due to DPT were also infrequent (Kuniyoshi et al., 2022). Some studies indicated that the false labeling of β-lactam anaphylactic reactions could be attributed to the virus infection (Caubet et al., 2011; Mori et al., 2015). Studies reported that direct DPT in children with a history of β-lactam anaphylaxis may be a safe and accurate strategy (Mill et al., 2016; Vyles et al., 2017a; Ibáñez et al., 2018; Labrosse et al., 2018). A study evaluated the frequency of severe adverse reactions after a direct DPT in patients with reported historical allergies to penicillin or other β-lactam antibiotics (Cardoso-Fernandes et al., 2021). The result of the study indicated that severe reactions due to DPT are infrequent and the superior safety of the DPT method supports its application in the diagnosis of penicillin anaphylaxis to contribute to ensuring the correct use of antibiotics, minimizing drug-induced risks, and improving clinical treatment outcomes.

However, DPT reproduces not only hypersensitivity symptoms but also any other adverse clinical manifestation. Some patients do not like to be re-exposed to the drug. Thus, DPT may be harmful and should only be considered after balancing the risk–benefit ratio for the individual patient (Bousquet et al., 2008). In addition, the PPV of DPT may be lower than expected. Thus, a second DPT is suggested to be performed within a few weeks or months. A study suggested that the allergic result should be confirmed with a second DPT within a few weeks or months to remove false labeling of allergies and ensure the safe use of drugs (Moral et al., 2022).

### 5.2 Oral provocation test

The oral provocation test (oral challenge) is the method to determine whether a patient is allergic to the drug or not. A systematic review found two studies reporting a positive predictive value of skin tests in children of 36% and 33%, respectively. A skin test could lead to an inaccurate diagnosis. An oral provocation test was finally needed to confirm tolerance in most of these children. In immediate and non-immediate reactions, the gold standard procedure to determine acute β-lactam tolerance was the oral provocation test. Oral challenge used a therapeutic β-lactam dose and at least 1 h of observation; it was costly and time-consuming Confino-Cohen et al., 2017. In the case of mild non-immediate reactions in children, skin tests were less commonly used, and oral provocation tests were a safe procedure (Moral and Caubet, 2017; Graham et al., 2018). The oral provocation test is formally contraindicated if there is a history of severe cutaneous adverse reactions (Felix and Kuschnir, 2020). In some studies, the evaluation of the direct oral provocation test was performed excluding high-risk patients (Iammatteo et al., 2019; Kuruvilla et al., 2019; Mustafa et al., 2019).

The oral provocation test is considered accurate with high positive and negative predictive values. A direct oral provocation test without a previous skin test has been increasingly used in patients, especially children with a history of mild, non-immediate reactions to β-lactam. In the case of mild non-immediate reactions in children, skin tests were less common and oral provocation tests were a safe procedure (Felix and Kuschnir, 2020). A study evaluated 119 children with a history of mild, non-immediate cutaneous reactions induced by β-lactam through direct oral provocation. Only four (3.4%) reacted with urticaria during oral provocation, and there was no severe reaction (Vezir et al., 2016). Further studies, including those of various populations and age groups, are needed to enable a stronger recommendation in this regard.

## 6 Conclusion

β-lactam antibiotics, including penicillin and cephalosporin, are common causes of drug hypersensitivity reactions in children. The β-lactam antibiotic skin test is widely used to predict anaphylactic reactions before medication. However, multiple studies highlighted the suboptimal diagnostic value of skin tests in children; positive results of skin tests were more often encountered in pediatrics than in adults. In fact, most children with reported β-lactam allergies are not allergic, which leads to the use of broad-spectrum antibiotics, additional costs, and significantly increased drug resistance and complications.

Given the limitations of β-lactam antibiotic skin tests, drug provocation tests, and oral challenges, these were the current standards in the management of pediatric β-lactam allergies because there are no standardized protocols at present. Direct drug provocation tests and oral challenges by skipping skin tests in appropriate patients were gaining acceptance as delabeling strategies. These strategies would learn from skin tests in mutual complementarity.

## Author contributions

LZ and WL provided ideas for the manuscript and reviewed the manuscript. CG consulted references and wrote the manuscript. BM provided advice for further modifications to this manuscript. All authors contributed to the article and approved the submitted version.
